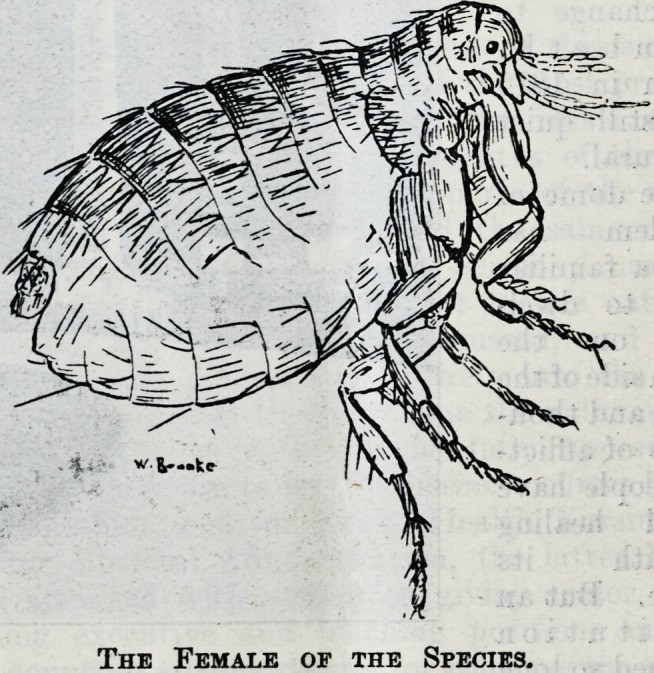# A Sprightly Acrobat

**Published:** 1924-11

**Authors:** 


### A SPRIGHTLY ACROBAT.

### THE UBIQUITOUS PULEX IRRITANS.

JUST as most young doctors have never seen a case of small-pox, so many nicely brought up young people have never seen a flea. This is not to say that the flea is growing extinct. The growth of cleanly habits may have banished him from many houses, but we are all, King or peasant, liable to meet him unexpectedly, to our certain discomfiture, and few are they who pass through life without, at all events, an occasional battle with *pulex irritans.*

LARVA OF THE FLEA.

Even royal personages have had their little troubles of this kind. In former days, exhibitions of "Trained Fleas" were not uncommon, and a little tale was once whispered concerning a princess who was present at one of them and took so much interest in it that when one of the little actors escaped she allowed it to be recaptured from her dress. Her gracious condescension was ill-rewarded, for when the errant one was returned to his supposed proprietor the great lady's chagrin was great at the emphatic statement that this was no educated insect, but the princess's own property. If rumour speak correctly the man might well know his own, it being said that the jumping legs of all "trained" fleas were amputated before their education began.

### THE GENESIS OF THE BLOODSUCKER.

Most people know how the insect gets its living, and have, at least, heard how its polished, hard and horny skin glides through the fingers itching for vengeance. The creature sits still, is pounced upon, and is nowhere to be seen. There are naturalists who know all about fleas, and can tell us their life history from its start to its often sudden and tragic finish. They will tell us of a dozen sticky whitish eggs laid in the fur of an animal or perhaps oftener in the dust—for preference a dust mixed with feathers or a little animal refuse, dried bits of skin, etc. Then comes the hatching of a tiny worm or caterpillar which, when full grown, is like an eighth of an inch of very fine reddish hair till the microscope shows that it is segmented, has tiny antennæ and two minute hooks on its tail. They will tell us further of the cocoon spun by this tiny silkworm, enclosing a chrysalis, legless and without power of movement, but in shape something like the mature insect. From this in a few days emerges the better-known form of the complete flea.

### THE BENEFIT OF THE CONTRACT.

There is a Rabbinical legend which professes to account for the origin of human parasites of the insect type. When Noah's Ark, with its precious cargo of all surviving animal life, was drifting round among the rocky mountains of Asia Minor, it sprang a leak. Noah, with his weighty responsibilities and not being much of a sailor, was much worried till the serpent came to the rescue and offered to stop the leak if Noah would undertake to feed him with human flesh when the voyage was over. The present apparently seemed to Noah more important than the future and the bargain was struck; the serpent insinuated his tail into the leak and the trouble was over. Then came the end of the voyage and the presentation of the serpent's little bill. It was a serious dilemma. Eight persons would not feed even one serpent for long. In his trouble Noah consulted the Archangel Gabriel, who advised burning the serpent and scattering his ashes in the air. This was done, and the ashes became fleas, flies and even worse, and went promptly to work to claim their respective shares of the benefits of the contract.

### AN INSIDIOUS RELATION.

In the West Indies there lives an unpleasing relative of the flea which constantly and ardently longs to bury itself under the skin that lines some human toenail. Once settled in such happy regions, the Chigo (or Jigger as it is familiarly called) swells with satisfaction and much easily assimilated food until it reaches the size of a small pea. Should it be left long enough to deposit its eggs the result might be a troublesome and even a dangerous wound.

THE FEMALE OF THE SPECIES.

Hence in those regions mothers have the excitement of a nightly "Jigger-hunt," the quarry, when found, being dug out with a sharp needle. Should one be overlooked till it reaches maturity, it requires to be very skilfully removed lest an egg be left behind. To make certain, the custom is to rub red pepper into the opening. Should an egg hatch, the patient has the additional joy of receiving a drop of spirits of turpentine in the wound—that, at all events, has been the received treatment. It might be supposed that the recipient of the Chigo's attentions would recognise and frustrate them at once, but the invader's manners are insinuating and even charming, only a pleasant titillation being excited by its intrusion.

To Catch and Kill.

As to dealings with our better known hero, an impatient adviser says, " Catch it and kill it," which is all very well for an isolated specimen. But if we place ourselves in the position of a well-known naturalist, who was once placed with a broken leg in a room which had been shut up for some years, and inhabited by an ever-multiplying horde of the industrious ones, we can see that there may be times when more radical measures are advisable. First, it will be necessary to put a check on the future supply ; and herein lies the value of a knowledge of the early life and habits of the insect. All dust will, of course, be removed. Floors will be scrubbed with carbolic or other strong disinfectant, while curtains, bed-clothing, pillows, and furniture will be brushed and beaten out of doors. Then Keating's insect powder, black or red pepper, may be sprinkled wherever dust is likely to accummulate, and one of the following mixtures painted into floor cracks, etc. : 1. Thirty parts of unpurified petroleum (paraffin) in one thousand parts of water. 2. Two ounces of solution of ammonia, one dram of saltpetre and one ounce of shaving soap mixed with a quart of soft water. Either of these mixtures is likely to have much the same effect upon *pulex irritans* as holy water is reputed to have upon " old Clootie."

## Figures and Tables

**Figure f1:**
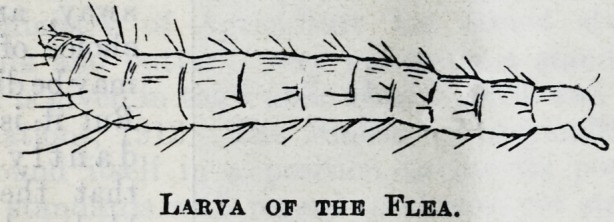


**Figure f2:**